# Sesquiterpene Lactones Isolated from *Centaurea cineraria* L. subsp. *cineraria* Inhibit the Radicle Growth of Broomrape Weeds

**DOI:** 10.3390/plants13020178

**Published:** 2024-01-09

**Authors:** Jesús G. Zorrilla, Michele Innangi, Antonio Cala Peralta, Gabriele Soriano, Maria Teresa Russo, Marco Masi, Mónica Fernández-Aparicio, Alessio Cimmino

**Affiliations:** 1 Department of Chemical Sciences, University of Naples Federico II, Complesso Universitario Monte S. Angelo, Via Cintia, 80126 Naples, Italy; jesus.zorrilla@uca.es (J.G.Z.); gabriele.soriano@unina.it (G.S.); mariateresa.russo2@unina.it (M.T.R.); alessio.cimmino@unina.it (A.C.); 2 Allelopathy Group, Department of Organic Chemistry, Facultad de Ciencias, Institute of Biomolecules (INBIO), University of Cadiz, C/Avenida República Saharaui, s/n, 11510 Puerto Real, Spain; antonio.cala@uca.es; 3 EnvixLab, Department of Biosciences and Territory, University of Molise, Contrada Fonte Lappone, 86090 Pesche, Italy; michele.innangi@unimol.it; 4 Department of Plant Breeding, Institute for Sustainable Agriculture (IAS), CSIC, Avenida Menéndez Pidal s/n, 14004 Córdoba, Spain

**Keywords:** parasitic weeds, *Orobanche*, *Phelipanche*, allelochemicals, cnicin, sesquiterpene lactones, sustainable crop protection

## Abstract

The plant *Centaurea cineraria* L. subsp*. cineraria* has been investigated as a potential source of inhibitors of broomrape radicle growth. The latter are weeds that pose a threat to agriculture and for which there are few methods available for the control of infestations. Four sesquiterpene lactones have been isolated from *C. cineraria* L. subsp*. cineraria* aerial parts and identified as isocnicin, cnicin, salonitenolide, and 11β,13-dihydrosalonitenolide using spectroscopic, spectrometric, and optical methods. Salonitenolide and 11β,13-dihydrosalonitenolide have been isolated for the first time from this plant. Tested at 1.0–0.1 mM against the broomrape species *Phelipanche ramosa*, *Orobanche minor*, *Orobanche crenata*, and *Orobanche cumana*, isocnicin, cnicin, and salonitenolide demonstrated remarkable inhibitory activity (over 80% in most of the cases) at the highest concentrations. Structure-activity relationship conclusions indicated the significance of the α,β-unsaturated lactone ring. In addition, the synthetic acetylated derivative of salonitenolide showed the strongest activity among all compounds tested, with inhibitions close to 100% at different concentrations, which has been related to a different lipophilicity and the absence of H-bond donor atoms in its structure. Neither the extracts nor the compounds exhibited the stimulating activity of broomrape germination (induction of suicidal germination). These findings highlight the potential of *C. cineraria* to produce bioactive compounds for managing parasitic weeds and prompt further studies on its sesquiterpene lactones as tools in developing natural product-based herbicides.

## 1. Introduction

Plants are able to produce a wide range of secondary metabolites in response to both biotic and non-biotic stresses, as well as for allelopathic interactions. The biological phenomenon of allelopathy is the direct or indirect effect of chemicals released by one organism on the physiological processes of other neighboring organisms [1]. These allelochemicals can be directly used or serve as nature-inspired models for the development of new herbicides as alternatives to synthetic pesticides [2]. The ongoing search for new potential herbicide models is imperative due to the continuous adaptability of weeds, leading to their evolution and acquisition of resistance to traditional herbicides or efficacy to resistant crop biotypes [3]. Parasitic weeds pose a particular threat, with their impact manifesting after years of seed dormancy in fields. Upon germination, they rapidly establish connections with host vasculature for water and nutrient supplies through the development of haustoria [4]. Broomrape weeds (Orobanchaceae), comprising various *Orobanche* and *Phelipanche* species, represent a great risk to important agricultural crops. Noteworthy examples include Fabaceae (affected by *Orobanche crenata*, *Orobanche foetida*, *Orobanche minor*, *Phelipanche ramosa*, or *Phelipanche aegyptiaca*), Apiaceae (affected by *O. crenata*, *O. minor*, or *P. ramosa*), Solanaceae (affected by *O. minor* or *P. ramosa*), and sunflower (*Helianthus annuus*) crops, the latter with a specific affection from *Orobanche cumana* [5,6]. The lack of efficient methods for broomrape control can lead to significant crop losses, and there is a prediction of an increasing impact of Orobanchaceae worldwide [5,7,8,9].

In the pursuit of sustainable methods to control broomrapes, great attention is directed towards studying plants whose extracts or specialized metabolites exhibit phytotoxicity or germination-inducing activity (suicidal germination) [10,11,12]. Recent studies have demonstrated positive outcomes with plants and their secondary metabolites by studying safflower (*Carthamus tinctorius*), maize (*Zea mays* L.) or the invasive plant *Conyza bonariensis* [11,13,14] from this perspective. However, only a limited number of local species have been explored as sources of allelochemicals against parasitic weeds, underscoring the potential for significant discoveries to address parasitic infestations from the perspective of allelopathy. Among these plants, those belonging to the *Centaurea cineraria* species are still unexplored for their potential in parasitic plant research. The genus *Centaurea* comprises flowering plants in the Asteraceae family, some of which are considered invasive weeds [15,16]. In particular, *C. cineraria* is characterized by tomentose leaves and flower heads with purplish flowers, whose preferred habitat is carbonate cliffs near the sea [17]. *C. cineraria* belongs to a species complex not yet fully understood from an evolutionary point of view that has differentiated around the Tyrrhenian Sea over the last 250,000 years, representing an extremely interesting group of plants due to their rapid evolution [17]. To the group of taxa afferent to *C. cineraria* also belong some subspecies endemic to Italy, including *C. cineraria* L. subsp. s*irenum* (Lacaita) Pignatti & Iamonico ex Iamonico & Del Guacchio, endemic to the Li Galli archipelago off the Amalfi Peninsula in Campania, and *C. cineraria* L. subsp. *circae* (Sommier) Cela Renz. & Viegi, endemic to the Circeo area in Latium [18,19]. The nominal subspecies is present in Italy in the regions bordering the Tyrrhenian Sea from Campania to Calabria (excluding Sicily), but there are also populations in Tunisia [17]. The extracts obtained from different parts of various *Centaurea* spp. showed a wide range of bioactivity, including seed growth inhibition [20,21,22]. Furthermore, diverse bioactive sesquiterpene lactones, natural products that possess documented activities against parasitic weeds, have been reported from *Centaurea* spp. [23,24,25]. Thus, *C. cineraria* L. subsp. *cineraria* (Asteraceae) has been selected and evaluated in this study for its potential to produce allelochemicals that can be used for the development of novel bioherbicides to manage parasitic weeds. This manuscript focuses on the isolation of secondary metabolites from this plant and on their chemical and biological characterization. Furthermore, structure-activity relationship (SAR) observations have been defined to identify the most suitable structural features for generating the target activity and for designing derivatives to optimize their efficacy [26,27]. The overall aim of this study is to provide natural compounds that can be used as model compounds in new bioherbicide formulations for parasitic weed management strategies. These preparations could be proposed as alternatives to chemical pesticides to increase efficacy and selectivity against broomrapes, as well as to overcome environmental problems and ensure food safety.

## 2. Results

### 2.1. Plant Extraction

**Figure 1:**
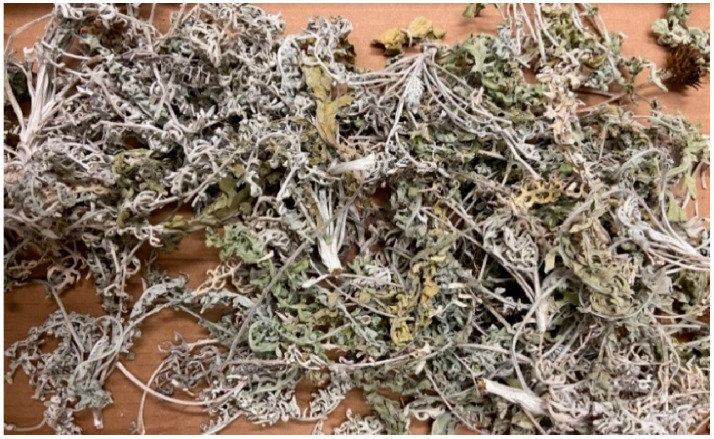
Dried *Centaurea cineraria* L. subsp. *cineraria* aerial parts employed in this study.

Dried *C. cineraria* L. subsp. *cineraria* aerial parts (Figure 1) were minced and macerated with an hydroalcoholic solution (H_2_O/methanol 1/1, *v*/*v*) for 24 h. After filtration, the obtained solution was extracted employing solvents with varying degrees of increasing polarity. Thus, 170.2 mg were obtained using *n*-hexane, 984.2 mg using dichloromethane (CH_2_Cl_2_), and 634.1 mg using ethyl acetate (EtOAc).

### 2.2. Bio-Activity-Guided Purification of Secondary Metabolites

**Figure 2:**
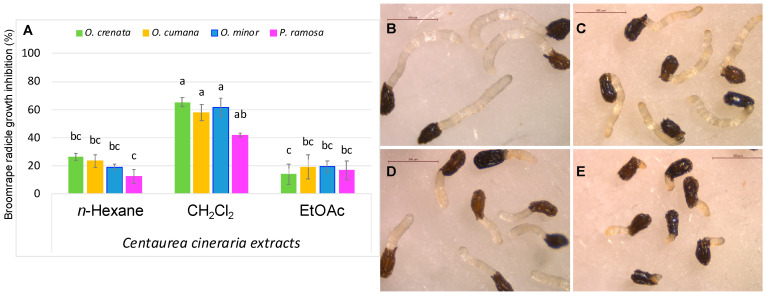
(**A**) Extracts effects on radicle growth of broomrape (*P. ramosa*, *O. minor*, *O. cumana*, and *O. crenata*) obtained by extraction with *n*-hexane, CH_2_Cl_2_, and ethyl acetate of *C. cineraria* aerial parts. Different letters show a significant difference between compounds by Tukey’s multiple comparison test (*p* < 0.05). Error bars represent the standard error of each mean (n = 3). (**B**–**E**) Illustrative pictures of *O. cumana* (**B**,**D**) and *O. minor* (**C**,**E**) treated with control (**B**,**C**) and CH_2_Cl_2_ extract (**D**,**E**).

Preliminary thin layer chromatography (TLC) analyses indicated the presence of a main metabolite in the CH_2_Cl_2_ extract, which was similarly detected in a lower amount in the EtOAc extract. The capacity of the extracts obtained from *C. cineraria* aerial parts to induce suicidal seed germination or to inhibit radicle growth was analysed using independent bioassays on four broomrape species seeds (*P. ramosa*, *O. minor*, *O. cumana*, and *O. crenata*). The tested concentrations were 100 and 10 μg/mL of dry extract in distilled water. For the radicle inhibition bioassay, the CH_2_Cl_2_ extract applied at 100 μg/mL showed marked activity in all the broomrape species (Figure 2).

Specifically, higher activity levels were observed for *O. crenata*, *O. cumana*, and *O. minor* (65.4 ± 3.6%, 57.9 ± 5.9%, and 61.5 ± 6.7% of inhibition, respectively), and 41.8 ± 1.6% in the case of *P. ramosa*, in comparison with negative controls. On the other hand, the *n*-hexane and EtOAc extracts produced lower growth inhibitions (ranging from 12.3 ± 5.1% to 26.3 ± 2.4%, Figure 2) in comparison with the negative control radicles. For the germination induction bioassay, no activity was observed against any of the four broomrape species studied.

**Figure 3:**
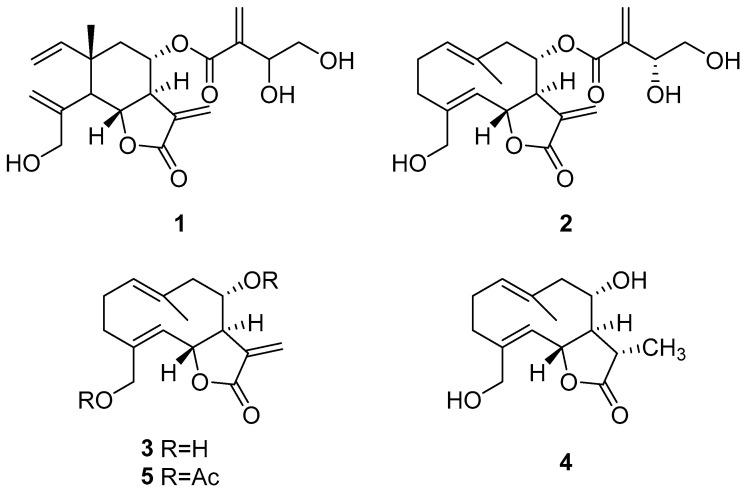
Structures of isocnicin (**1**), cnicin (**2**), salonitenolide (**3**), 11β,13-dihydrosalonitenolide (**4**), and the synthetic derivative 8,15-*O*,*O′*-diacetylsalonitenolide (**5**).

To isolate the metabolites exhibiting phytotoxicity on radicle growth, the most active extract (CH_2_Cl_2_) was purified by chromatography, resulting in the obtaining of four pure compounds identified as isocnicin, cnicin, salonitenolide, and 11β,13-dihydrosalonitenolide (**1**–**4**, Figure 3). Their optical and spectroscopic data are in accordance with those previously reported [28,29,30]. The isolated compounds were sesquiterpene lactones described for the *Centaurea* genus [28,31,32], while this is the first report on the identification and isolation of salonitenolide (**3**) and 11β,13-dihydrosalonitenolide (**4**) from *C. cineraria*.

### 2.3. Bioassays against Broomrapes

**Figure 4:**
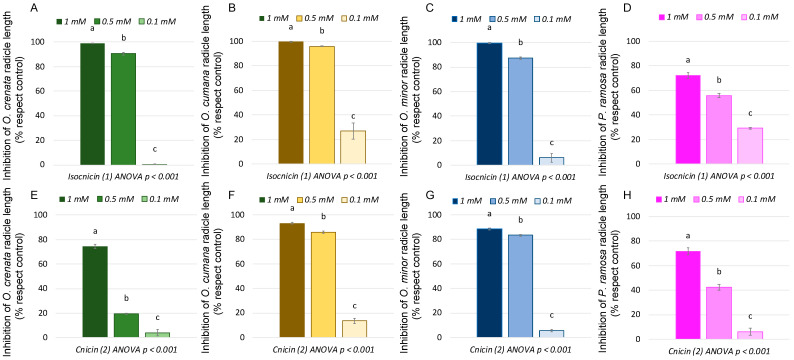
Growth inhibition induced by isocnicin (**1**, **A**–**D**) and cnicin (**2**, **E**–**H**) in the radicles of *O. crenata* (**A** and **E**), *O. cumana* (**B** and **F**), *O. minor* (**C** and **G**), and *P. ramosa* (**D** and **H**). Bars with different letters are significantly different according to the Tukey test (*p* < 0.05). Error bars represent the standard error of the mean.

**Figure 5:**
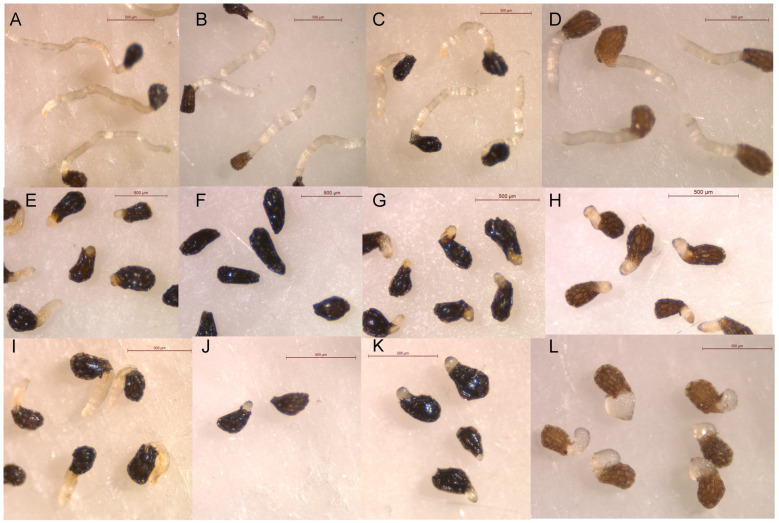
Control treatments (**A**–**D**), isocnicin (**1**, (**E**–**H**)), and cnicin (**2**, (**I**–**L**)) applied at 0.5 mM on radicles of *O. crenata* (**A**,**E**,**I**), *O. cumana* (**B**,**F**,**J**), *O. minor* (**C**,**G**,**K**), and *P. ramosa* (**D**,**H**,**L**).

A first screening with isocnicin (**1**) and cnicin (**2**) at a concentration range of 1.0–0.1 mM showed that they are active against *O. crenata*, *O. cumana*, *O. minor*, and *P. ramosa* (Figure 4). Notably, they displayed remarkable activity at the highest tested concentrations (1 and 0.5 mM), but there was a significant loss of activity observed at 0.1 mM. Specifically, at 1 mM, isocnicin (**1**) achieved nearly complete inhibition of the radicle growth of *O. crenata* (99.0 ± 0.2%), *O. cumana* (99.3 ± 0.5%), and *O. minor* (99.5 ± 0.5%). Substantial inhibition levels were also observed at 0.5 mM (90.6 ± 1.1%, 95.8 ± 0.3%, and 87.4 ± 0.8%, respectively). Figure 5E–H shows how, besides the inhibition of radicle growth, isocnicin (**1**) induced darkening of the broomrape radicles when applied at 1 and 0.5 mM. In the case of cnicin (**2**), similar trends were observed against *O. cumana* and *O. minor*, but there was a decrease in activity from 0.5 mM against *O. crenata* and *P. ramosa* (Figure 4). The darkening effect on broomrape radicles induced by isocnicin (**1**) was not observed following cnicin treatments (Figure 5I–L), nor in the control radicles (Figure 5A–D). Consequently, inhibitory activity was found for both types of structures (elemanolide and germacranolide). As reported in the literature, the biological activity of sesquiterpene lactones is strongly related to the lactone ring [33]. However, the improved activity observed for isocnicin (**1**), especially against *O. crenata* (Figure 4), should be attributed to a structural advantage of the elemanolide scaffold when compared to germacranolides.

**Figure 6:**
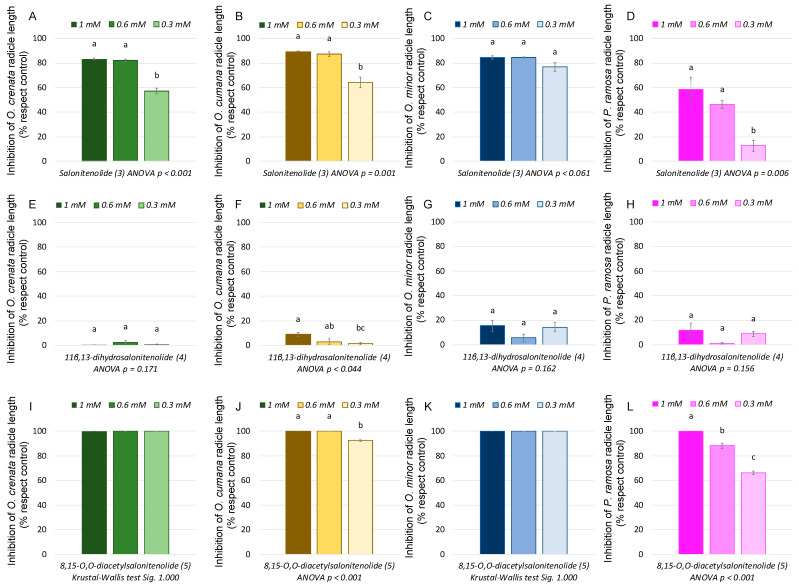
Growth inhibition induced by salonitenolide (**3**, **A**–**D**), 11β,13-dihydrosalonitenolide (**4**, **E**–**H**), and 8,15-*O*,*O*-diacetylsalonitenolide (**5**, **I**–**L**) in radicles of *O. crenata*, *O. cumana*, *O. minor*, and *P. ramosa*. Bars with different letters are significantly different according to the Tukey test (*p* < 0.05). Error bars represent the standard error of the mean.

**Figure 7:**
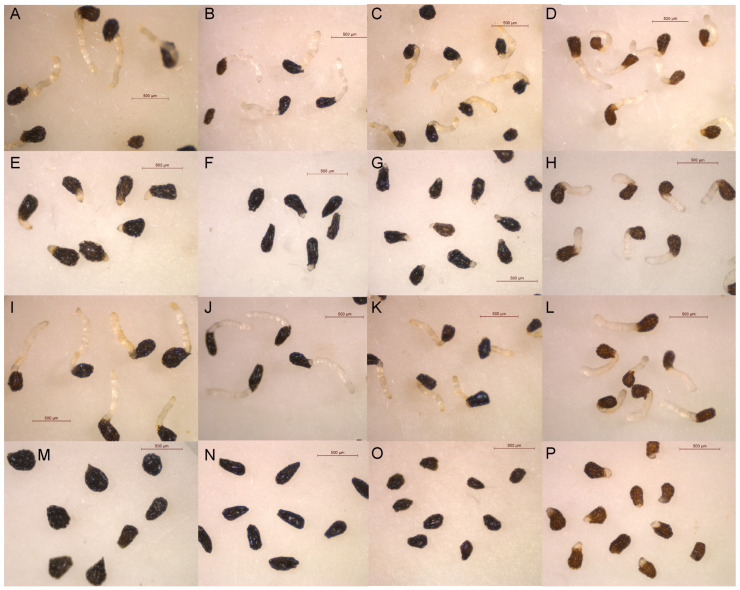
Treatments with control (**A**–**D**), salonitenolide (**3**, (**E**–**H**)), 11β,13-dihydrosalonitenolide (**4**, (**I**–**L**)), applied at 1 mM, and 8,15-*O*,*O*-diacetylsalonitenolide (**5**, (**M**–**P**)) on radicles of *O. crenata* (**A**,**E**,**I**,**M**), *O. cumana* (**B**,**F**,**J**,**N**), *O. minor* (**C**,**G**,**K**,**O**), and *P. ramosa* (**D**,**H**,**L**,**P**).

Salonitenolide (**3**) and 11β,13-dihydrosalonitenolide (**4**) activities in the same type of bioassay were evaluated, adapting the range of concentrations to 1.0–0.3 mM in accordance with the aforementioned results. The activity profiles obtained were notably contrasting, showing compound **3** high inhibition levels in most of the cases (Figure 6A–D), unlike compound **4**, which showed poor activity, always below 15.4 ± 4.6% (Figure 6E–H and Figure 7A–L). Specifically, the inhibitions achieved by salonitenolide (**3**) against *O. crenata* were 83.4 ± 0.7%, 82.2 ± 0.7%, and 57.1 ± 2.4%, against *O. cumana* by 89.4 ± 0.4%, 87.3 ± 1.9%, and 64.2 ± 4.3%, against *O. minor* by 84.6 ± 1.4%, 84.5 ± 0.4%, and 76.8 ± 3.5%, and against *P. ramosa* by 58.5 ± 10.1%, 46.2 ± 3.2%, and 12.7 ± 4.6% (at 1 mM, 0.6 mM, and 0.3 mM, respectively) (Figure 6A–D). Aiming at providing insights into the bioactivity of salonitenolide (**3**), its acetylated derivative 8,15-*O*,*O’*-diacetylsalonitenolide (**5**) was synthetically obtained and tested in bioassay. As a result, compound **5** showed the strongest activity among all the isolated compounds from *C. cineraria*, completely inhibiting the growth of radicles of *O. crenata* and *O. minor* at all concentrations tested, inhibiting the *O. cumana* radicles by 100 ± 0.0%, 100 ± 0.0%, and 92.5 ± 0.6%, and the *P. ramosa* radicles by 100 ± 0.0%, 88.3 ± 2.2%, and 66.4 ± 1.3%, at respective concentrations of 1, 0.6, and 0.3 mM (Figure 6I–L and Figure 7M–P).

## 3. Discussion

From a structural perspective, two distinct types of sesquiterpene lactones were isolated. Isocnicin (**1**) features an elemanolide structure, while compounds **2**–**4** are germacranolides. A few specific lactones produced by *Centaurea* spp. have been identified as phytochemicals, with cnicin (**2**) being widely studied in different fields, including reports on its phytotoxicity [34,35]. Consequently, the inhibitory activity of the isolated compounds against broomrape radicle growth was evaluated in bioassays. The results obtained allowed us to discuss some structure-activity relationships. The key one is the importance of the double bond in the α,β-unsaturated lactone ring of the sesquiterpene lactones in the inhibition of broomrape, given the poor activity of 11β,13-dihydrosalonitenolide (**4**) in comparison with the results of the other compounds tested. It could be highlighted how a previous study showed that an 11,13-dihydro sesquiterpene lactone could be active in bioassays with broomrapes [36]. When comparing parameters like lipophilicity and the total rotable bonds, H-bond donors, and H-bond acceptors, the similar values for compounds **3** and **4** (Table 1) may indicate that these are not directly correlated with the loss of activity by compound **4**.

The second SAR conclusion is related to the acetylation of the hydroxyl groups, which represents a relevant improvement in the inhibitory activity as observed for compound **5** in comparison with salonitenolide (**3**), leading to a significant increment of the inhibition, especially at the lowest concentrations (Figure 6). This improvement could be explained by a better solubility of the compound within hydrophobic environments like the lipid bilayers of membranes, as justified by the lipophilicity expressed by the partition coefficient values calculated by the clog *P* algorithm for compounds **1**–**5** (Table 1). Even though compounds **1**–**5** accomplish the Lipinskii rule for the optimal clog *P* value for herbicides (≤3.5) [37], compound **5** has a notably different value (2.45) in comparison to those of compounds **1**–**4** (0.37–0.64), which could therefore justify the different level of activity. Moreover, the improved activity of compound **5** could be related to the absence of H-bond donor atoms in its structure, unlike the structures of compounds **1**–**4** (Table 1). Regarding the activity for induction of broomrape germination, even though the *C. cineraria* organic extracts were inactive as inductors of broomrape germination, a bioassay with compounds **1**–**5** was carried out on the seeds of *P. ramosa*, *O. minor*, *O. cumana*, and *O. crenata* to test their suicidal germination induction activity in the range of concentrations described above for radicle inhibition. Null activity was obtained in all the cases, proving that the null activity of the extracts was not due to other potential minor metabolites in the extract interfering with the bioactivity, as well as that the acetylation of compound **3** is not a modification able to change the behavior of this compound for suicidal germination activity.

The results herein presented fit into the context of the biology and management of parasitic weeds. The germination of broomrape seeds is commonly stimulated by specific compounds exuded by potential host plants in their surroundings. The family of phytohormones named strigolactones represents the most studied structures, though diverse sesquiterpene lactones produced by plants have been discovered as potent elicitors of germination of some broomrape species, being inactive on the germination of other broomrape species, with the case of costunolide and dehydrocostus lactone produced by sunflower being one of the most representative [10,23]. Thus, the study of sesquiterpene lactones also focused on the interest of parasitic weed research, covering topics like mechanisms, biosynthesis, and the use of materials for the development of bioactive derivatives against parasitic weeds [38,39]. Regarding the inhibition of parasitic weeds, few references are available in the literature, mainly the discovery of inuloxins as inhibitors of the germination of *O. crenata* and *Cuscuta campestris* [40]. Thus, this study provides different sesquiterpene lactones of interest for the development of tools based on natural products for the management of broomrape weeds. This includes the elemanolide isocnicin (**1**), two natural germacranolides (cnicin **2**, and salonitenolide **3**), and the highly active synthetic derivative 8,15-*O*,*O′*-diacetylsalonitenolide (**5**). It may be noted that germacranolides are a subgroup of sesquiterpene lactones with diverse biological activities of agronomical and pharmacological interest, and from which it is worth highlighting the capacity of some of them to stimulate the germination of specific broomrape species [23,41,42]. Furthermore, taking into account that the active compounds contain an α,β-unsaturated lactone ring, they could be used as starting material for the synthesis of new strigolactone analogues following already reported strategies [43]. Previous reports on the synthesis of derivatives of cnicin would also encourage the study of additional derivatives with improved activities in bioassays with parasitic plants [44,45].

## 4. Materials and Methods

### 4.1. General Experimental Procedures

A Bruker 400 Anova Advance (Karlsruhe, Germany) spectrometer was used to record the proton nuclear magnetic resonance (^1^H NMR) at 400 MHz. CDCl_3_ was used as a solvent and internal standard. A digital polarimeter, the JASCO P-1010 (Tokyo, Japan), was used to measure the optical rotations. Liquid Chromatography/Mass Spectrometry-Time of Flight (LC/MS TOF) system AGILENT 6230B (Agilent Technologies, Milan, Italy) and High-performance liquid chromatography (HPLC) 1260 Infinity were used to perform Electrospray ionisation mass spectra (ESIMS). Column chromatography (CC) was performed using silica gel (Kieselgel 60, 0.063–0.200 mm, Merck, Darmstadt, Germany). Analytical and preparative silica gel plates (Kieselgel 60, F_254_, 0.25 and 0.5 mm, respectively, Merck, Darmstadt, Germany) and thin-layer chromatography (TLC) were performed. Spots Visualization was carried out by exposure to UV light (254 nm) and/or iodine vapors and/or by spraying first with 10% H_2_SO_4_ in MeOH and then with 5% phosphomolybdic acid in EtOH, followed by heating at 110 °C for 10 min. Sigma-Aldrich Co. (St. Louis, MO, USA) supplied all the reagents and the solvents.

### 4.2. Plant Material

*C. cineraria* L. subsp. *cineraria* was collected in February 2022 in the municipality of Massa Lubrense (Metropolitan City of Naples, Italy). The collection was carried out in a period far from flowering and fruiting, collecting the terminal part of leafy twigs from about 30 individuals, spaced 5–10 m apart to reduce the risk of sampling clonal individuals. In order to remove dust particles, distilled water was used to rinse plant material, which was then dried for a few days in the air at room temperature and ground in a blender. The identification of the plant material was conducted according to the flora of Italy [46] and confronted with reference material collected in the same area from the collection of Michele Guadagno kept at the herbarium of Pisa (PI-GUAD 012823, https://erbario.unipi.it/it/erbario/view?id=1615282 (accessed on 5 December 2023)). Broomrape (*Orobanche* and *Phelipanche*) seeds from four species of root parasitic weeds were used. Seeds of *O. crenata* were collected in 2019 from mature *Orobanche* plants infecting pea in Spain, and seeds of *O. cumana* were collected in 2017 from mature *Orobanche* plants infecting sunflower in Spain. *O. minor* seeds were collected in 2015 from *Orobanche* plants infecting red clover in France, and seeds of *P. ramosa* were collected in 2015 from *Phelipanche* plants infecting tobacco in France.

### 4.3. Purification and Identification Compounds ***1***–***4***

50 g of dried and minced *C. cineraria* L. subsp. *cineraria* were extracted by H_2_O/MeOH (1/1, *v*/*v*) under stirred conditions at room temperature for 24 h. The hydroalcoholic suspensions were centrifuged at 7000 rpm and extracted with *n*-hexane (2 × 200 mL), CH_2_Cl_2_ (2 × 200 mL), and, after removing methanol under reduced pressure, with EtOAc (2 × 200 mL). The residues of both extractions performed with the three solvents were combined, obtaining 85.2 mg (*n*-hexane), 492.1 mg (CH_2_Cl_2_), and 317.1 mg (EtOAc) of organic extracts.

The residue of the CH_2_Cl_2_ organic extract was purified by column chromatography using the eluent CHCl_3_/*i*-propanol (9/1, *v*/*v*), and eight homogeneous fractions (F1-8) were obtained. The residue of fraction F3 (34.7 mg) was further purified by TLC in the direct (eluted with CHCl_3_/*i*-propanol (95/5, *v*/*v*)) and reverse phases (eluted with acetonitrile/H_2_O (4/6, *v*/*v*)) phases, yielding a pure compound identified as salonitenolide (**3**, 18.1 mg). The residue of fraction F4 (8.6 mg) was further purified by reverse phase TLC using eluent ethanol/H_2_O (6/4, *v*/*v*), giving a pure compound identified as isocnicin (**1**, 6.6 mg). The residue (27.4 mg) of F5 was further purified using CH_2_Cl_2_/MeOH (9/1, *v*/*v*) as eluent, yielding a pure compound that was identified as 11β,13-dihydrosalonitenolide (**4**). The residue of F6 (277.8 mg) corresponded with a pure compound identified as cnicin (**2**).

Isocnicin (**1**): The ^1^H NMR spectrum (Figure S1) agreed with previously reported data [28]. ESIMS (+) *m*/*z*: 401 [M + Na]^+^ (Figure S2).

Cnicin (**2**): The ^1^H NMR spectrum (Figure S3) agreed with previously reported data [30]. ESIMS (+) *m*/*z*: 379 [M + H]^+^ (Figure S4). [α]_D_ +160.4 (*c* 0.10, MeOH), [α]_D_ +169.6 lit. [30].

Salonitenolide (**3**): ^1^H NMR spectrum (Figure S5) agreed with previously reported data [30]. ESIMS (+) *m*/*z*: 287 [M + Na]^+^ (Figure S6). [α]_D_ +195.9 (*c* 0.38, MeOH), [α]_D_ +199.4 lit. [30].

11β,13-Dihydrosalonitenolide (**4**): ^1^H NMR spectrum (Figure S7) agreed with previously reported data [29]. ESIMS (+) *m*/*z*: 267 [M + H]^+^ (Figure S8). [α]_D_ +101.2 (*c* 5.0, CHCl_3_), [α]_D_ +98 lit. [29].

### 4.4. Synthesis of 8,15-O,O′-Diacetylsalonitenolide (***5***)

3.0 mg of salonitenolide (**3**, 0.011 mmol) were dissolved in pyridine (20 μL), and acetic anhydride (20 μL) was added. The reaction was performed at room temperature overnight. MeOH was added to stop the reaction, and the azeotrope formed by benzene addition was evaporated under a N_2_ stream. The residue (3.2 mg) was purified by analytical TLC using CHCl_3_/*i*-propanol (97/3, *v*/*v*) as eluent, affording 8,15-*O*,*O’*-diacetylsalonitenolide (**5**) in 73% yield (2.8 mg, 0.008 mmol).

The obtaining of compound **5** as a diacetylated product of compound **3** was confirmed by comparison of their NMR and ESIMS spectroscopic data (Figures S5, S6, S9, and S10). Mainly, two new signals at δ 2.11 and 2.10 ppm with singlet multiplicity in the ^1^H NMR spectrum of compound **5** denoted the acetylation of two hydroxyl groups, which was also confirmed by the *m*/*z* value of 371 obtained in the ESIMS analysis of compound **5**, corresponding to a sodium adduct [M + Na]^+^ of a compound with a molecular weight of 348.

### 4.5. Growth Inhibition Assays on Broomrape Species

The phytotoxic activity of *C. cineraria* extracts and compounds was studied using growth inhibition bioassays on broomrape species, in which the candidate phytotoxin is applied to broomrape seeds induced to germinate by a two-step process required by broomrape to germinate, which consists of a first treatment of warm stratification called broomrape seed conditioning, followed by chemical stimulation with the synthetic germination stimulant GR24 [47]. Approximately 100 seeds of each parasitic species were individually placed on glass fiber filter paper (GFFP) discs (Whatman International Ltd., Maidstone, UK) of 9 mm-diameter and placed in Petri dishes. The filter paper was previously moistened with 50 μL of sterile distilled water. Petri dishes were sealed with parafilm and placed at 23 °C in the dark for 10 days to promote broomrape conditioning. Then, GFFP discs containing conditioned seeds were placed on a sterile sheet of filter paper to remove the water used to promote the conditioning and transferred to new 9 cm Petri dishes. *C. cineraria* compounds were first dissolved in dimethyl sulfoxide and then mixed with GR24 at the tested concentrations (1.0–0.1 mM). GFFP discs containing conditioned seeds of each broomrape species were applied to triplicate aliquots of each sample. Treatments containing GR24 and dimethyl sulfoxide were used as a control. The final concentration of dimethyl sulfoxide in all treatments was 1%. Treated seeds were incubated in the dark at 23 °C for 7 days, and then the radicle length was measured in each of the ten randomly chosen seedlings for each of the three replicate GFFP discs per treatment using a stereoscopic microscope (Leica S9i, Leica Microsystems GmbH, Wetzlar, Germany). The inhibition of radicle growth induced by each treatment was calculated relative to the average radicle growth of the control treatment.

### 4.6. Statistical Analysis and Calculations

Growth inhibition assays on broomrape species were performed using a completely randomized design. Percentage data were transformed using angular transformation to normalize data and stabilize variances. Analysis of variance (ANOVA) was conducted using SPSS software 27 (SPSS Inc., Chicago, IL, USA). The significance of mean differences among treatments was evaluated by Tukey’s multiple comparison test (*p* < 0.05).

Calculation of clog *P* values was performed using ChemOffice v20.1 (PerkinElmer, Waltham, MA, USA) using the appropriate tool in ChemDraw Professional, while the number of rotable bonds, H-bond acceptors, and H-bond donors was calculated using the SwissADME software [48,49,50].

## 5. Conclusions

This study describes for the first time a *Centaurea* species, namely *C. cineraria* L. subsp. *cineraria*, as a source of extracts and secondary metabolites as inhibitors of the radicle growth of broomrape weeds. Four sesquiterpene lactones were purified from the aerial parts of this plant and identified as isocnicin, cnicin, salonitenolide, and 11β,13-dihydrosalonitenolide. Isocnicin, cnicin, and salonitenolide, as well as the synthetic derivative 8,15-*O*,*O′*-diacetylsalonitenolide, exhibited significant inhibitory activity against diverse broomrape species, demonstrating inhibitions over 80% in most cases. Structure-activity relationship analyses emphasized the importance of the α,β-unsaturated lactone ring in their efficacy, and the greater efficacy of the synthetic acetylated derivative was attributed to enhanced lipophilicity and the absence of H-bond donor atoms in its structure. On the other hand, the *C. cineraria* organic extracts and the pure compounds did not induce suicidal germination of broomrape seeds. These findings highlight the potential of *C. cineraria* sesquiterpene lactones for developing natural product-based herbicides to combat parasitic weed infestations in sustainable crop protection strategies, and provides insights into the mechanisms involved in the germination of broomrapes. The significance of structural modifications in designing effective herbicides has been also remarked. In particular, 8,15-*O*,*O′*-diacetylsalonitenolide seems to be a promising compound to carry out other studies on its efficiency, degradation, and ecotoxicological profile. However, for its practical application in novel bioherbicide formulations, suitable amount of this compound will be required trough the development of its total stereoselective synthesis or its hemisynthesis, which can also be afforded starting from natural sesquiterpene lactones produced in relatively large amount. Furthermore, further research may cover the improvement of the biological activity of compounds at lower concentrations to minimize the required doses, by strategies like the obtaining of bioactive derivatives, encapsulation, or the study of mixtures, among others.

## Figures and Tables

**Table 1:** Clog *p* values and number of rotable bonds, H-bond donors, and H-bond acceptors of compounds **1**–**5**.

	Isocnicin (1)	Cnicin (2)	Salonitenolide (3)	11β,13-Dihydrosalonitenolide (4)	8,15-*O*,*O′*-Diacetylsalonitenolide (5)
**Clog *P***	0.37	0.63	0.61	0.64	2.45
**Rotable bonds**	8	6	1	1	5
**H-bond acceptors**	7	7	4	4	6
**H-bond donors**	3	3	2	2	0

## Data Availability

Data are contained within the article and Supplementary Materials.

## Supplementary Materials

The following supporting information can be downloaded at: https://www.mdpi.com/article/10.3390/plants13020178/s1. Figure S1. ^1^H NMR spectrum of isocnicin (1) recorded in CDCl_3_ at 400 MHz; Figure S2. ESI MS spectrum of isocnicin (1) recorded in positive mode; Figure S3. ^1^H NMR spectrum of cnicin (2) recorded in CDCl_3_ at 400 MHz; Figure S4. ESI MS spectrum of cnicin (2) recorded in positive mode; Figure S5. ^1^H NMR spectrum of salonitenolide (3) recorded in MeOD at 400 MHz; Figure S6. ESI MS spectrum of salonitenolide (3) recorded in positive mode; Figure S7. ^1^H NMR spectrum of 11β,13-dihydrosalonitenolide (4) recorded in CDCl_3_ at 400 MHz; Figure S8. ESI MS spectrum of 11β,13-dihydrosalonitenolide (4) recorded in positive mode; Figure S9. ^1^H NMR spectrum of 8,15-*O,O′*-diacetylsalonitenolide (5) recorded in CDCl_3_ at 400 MHz; Figure S10. ESI MS spectrum of 8,15-*O,O′*-diacetylsalonitenolide (5) recorded in positive mode.
