# Supplementary material for: Sesquiterpene Lactones Isolated from Centaurea cineraria L. subsp. cineraria Inhibit the Radicle Growth of Broomrape Weeds
Source: Plants (Basel). 2024 Jan 9;13(2):178. doi: 10.3390/plants13020178 (PMC10818712; doi:10.3390/plants13020178)
Supplement: Supplementary file 1 [file plants-13-00178-s001.zip › plants-2785825-supplementary.pdf]

Supporting Information

Sesquiterpene Lactones Isolated from *Centaurea cineraria* L. subsp. *cineraria* Inhibit the Radicle Growth of Broomrape Weeds

Jesús G. Zorrilla [1,2], Michele Innangi [3], Antonio Cala Peralta [2], Gabriele Soriano [1], Maria Teresa Russo [1], Marco Masi [1,*], Mónica Fernández-Aparicio [4,*] and Alessio Cimmino [1]

[1] Department of Chemical Sciences, University of Naples Federico II, Complesso Universitario Monte S. Angelo, Via Cintia, 80126 Naples, Italy; jesus.zorrilla@uca.es (J.G.Z.); gabriele.soriano@unina.it (G.S.); mariateresa.russo2@unina.it (M.T.R.); alessio.cimmino@unina.it (A.C.)
[2] Allelopathy Group, Department of Organic Chemistry, Facultad de Ciencias, Institute of Biomolecules (INBIO), University of Cadiz, C/Avenida República Saharaui, s/n, 11510 Puerto Real, Spain; antonio.cala@uca.es
[3] EnvixLab, Department of Biosciences and Territory, University of Molise, Contrada Fonte Lappone, 86090 Pesche, Italy; michele.innangi@unimol.it
[4] Department of Plant Breeding, Institute for Sustainable Agriculture (IAS), CSIC, Avenida Menéndez Pidal s/n, 14004 Córdoba, Spain
* Correspondence: marco.masi@unina.it (M.M.); monica.fernandez@ias.csic.es (M.F.-A.)

Supporting Information

Figure S1. ^{1}H NMR spectrum of isocnicin (**1**) recorded in $CDCl_3$ at 400 MHz 2

Figure S2. ESI MS spectrum of isocnicin (**1**) recorded in positive mode 2

Figure S3. ^{1}H NMR spectrum of cnicin (**2**) recorded in $CDCl_3$ at 400 MHz 3

Figure S4. ESI MS spectrum of cnicin (**2**) recorded in positive mode 3

Figure S5. ^{1}H NMR spectrum of salonitenolide (**3**) recorded in MeOD at 400 MHz 4

Figure S6. ESI MS spectrum of salonitenolide (**3**) recorded in positive mode 4

Figure S7. ^{1}H NMR spectrum of 11β,13-dihydrosalonitenolide (**4**) recorded in $CDCl_3$ at 400 MHz 5

Figure S8. ESI MS spectrum of 11β,13-dihydrosalonitenolide (**4**) recorded in positive mode 5

Figure S9. ^{1}H NMR spectrum of 8,15-*O,O′*-diacetylsalonitenolide (**5**) recorded in $CDCl_3$ at 400 MHz 6

Figure S10. ESI MS spectrum of 8,15-*O,O′*-diacetylsalonitenolide (**5**) recorded in positive mode 6

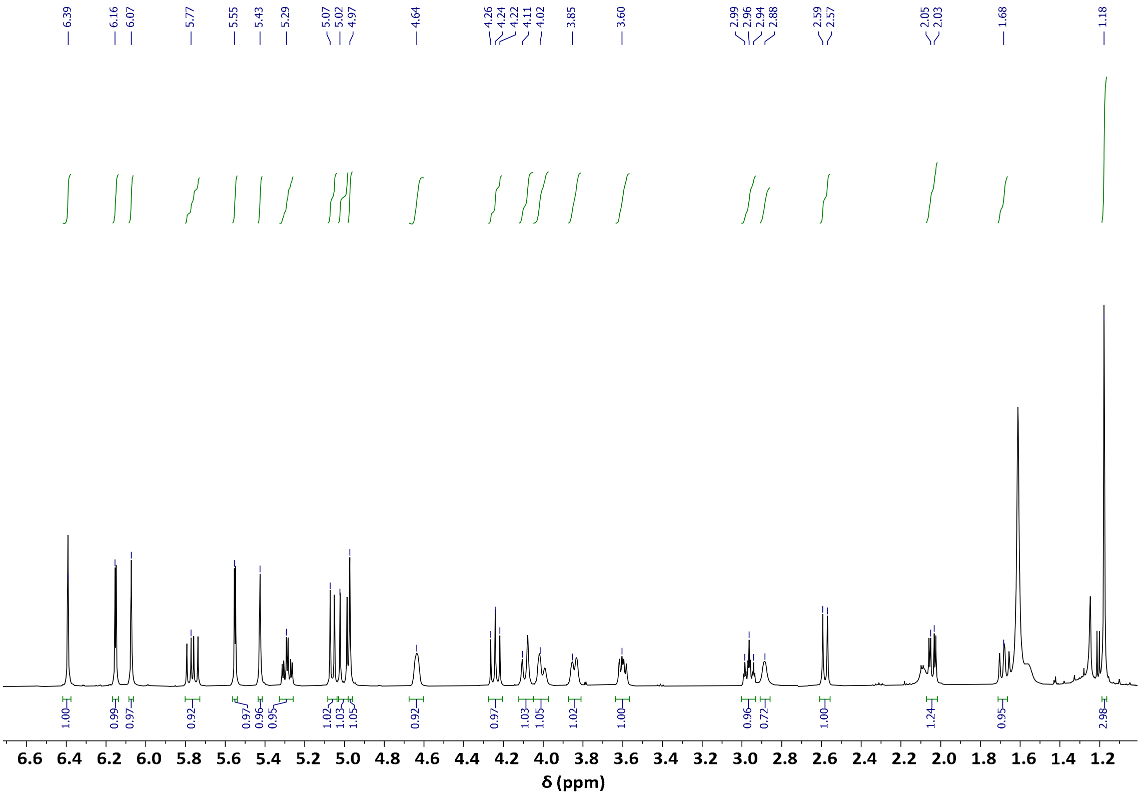

Figure S1. ^{1}H NMR spectrum of isocnicin (**1**) recorded in $CDCl_3$ at 400 MHz.

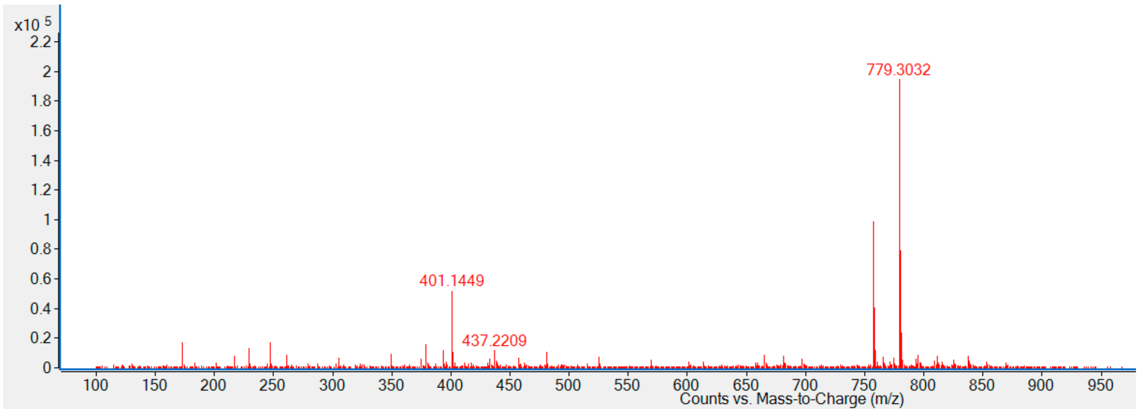

Figure S2. ESI MS spectrum of isocnicin (**1**) recorded in positive mode.

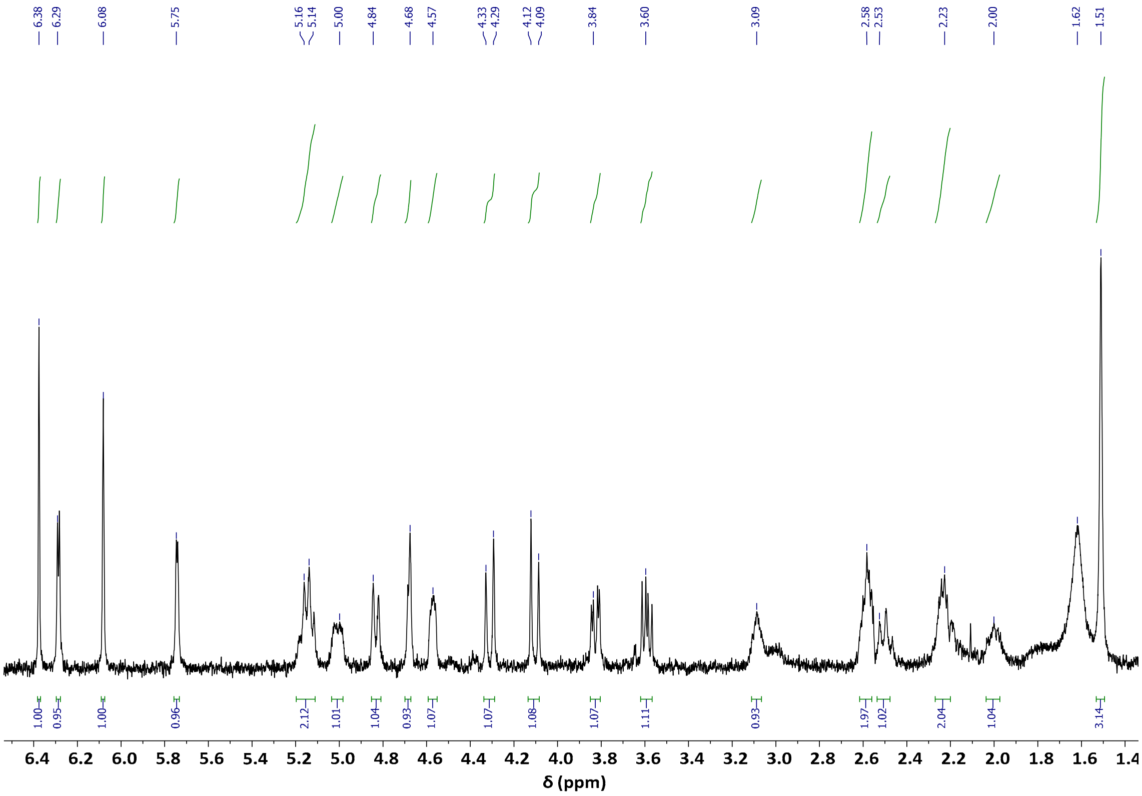

Figure S3. ^{1}H NMR spectrum of cnicin (**2**) recorded in $CDCl_3$ at 400 MHz.

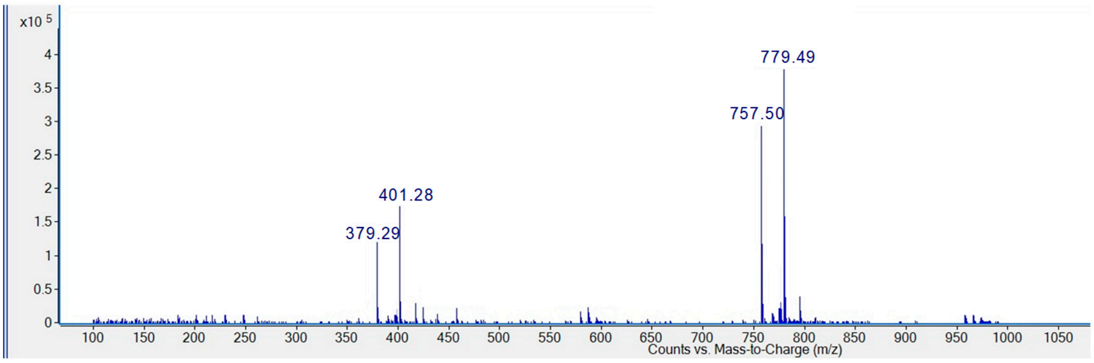

Figure S4. ESI MS spectrum of cnicin (**2**) recorded in positive mode.

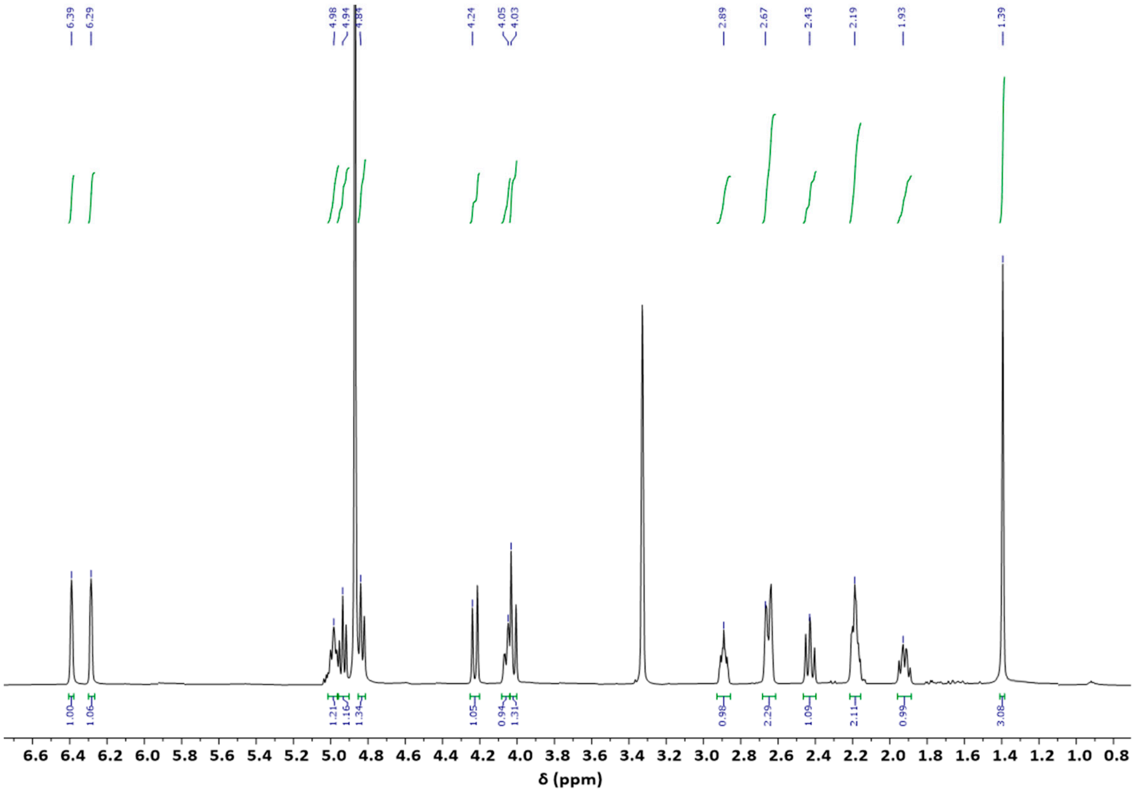

Figure S5. ^{1}H NMR spectrum of salonitenolide (**3**) recorded in MeOD at 400 MHz.

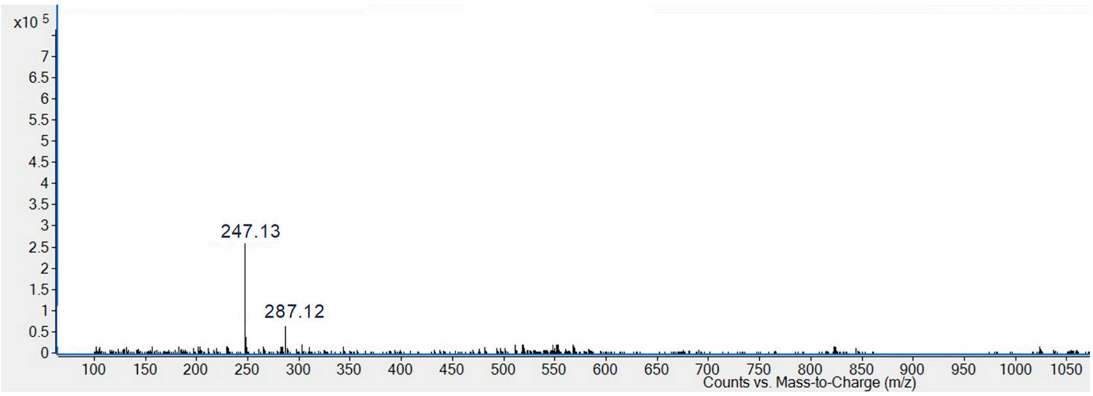

Figure S6. ESI MS spectrum of salonitenolide (**3**) recorded in positive mode.

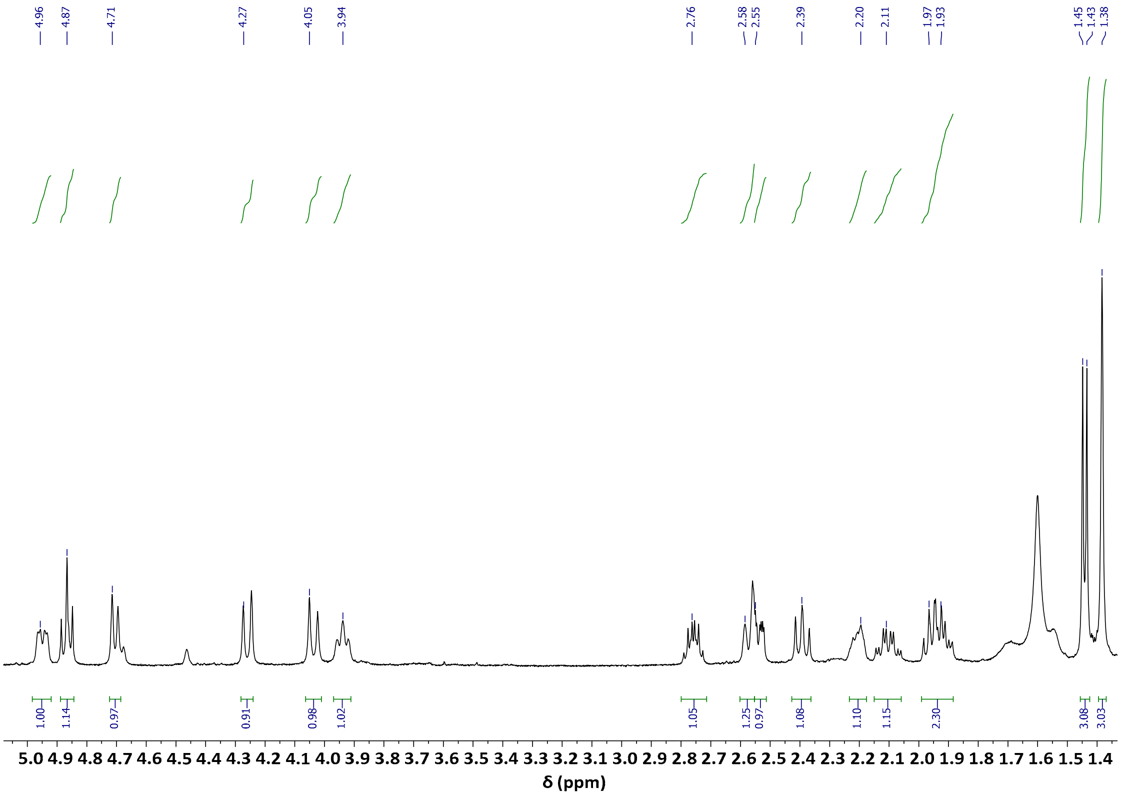

Figure S7. ^{1}H NMR spectrum of 11β,13-dihydrosalonitenolide (**4**) recorded in $CDCl_3$ at 400 MHz.

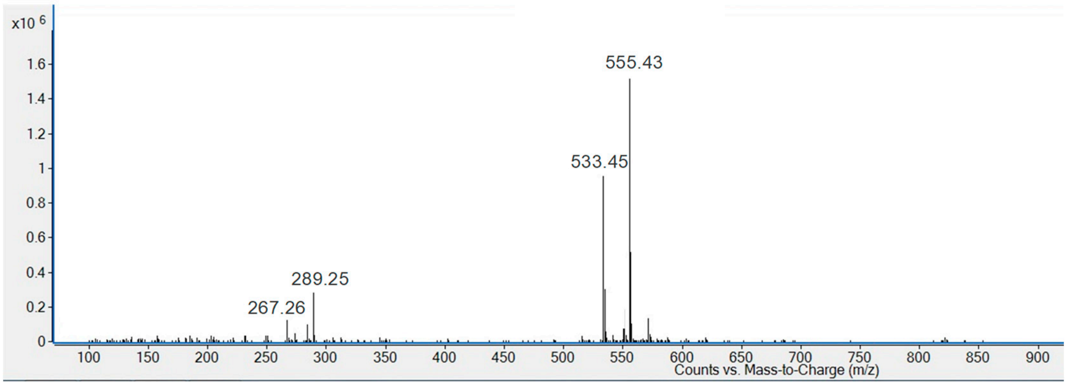

Figure S8. ESI MS spectrum of 11β,13-dihydrosalonitenolide (**4**) recorded in positive mode.

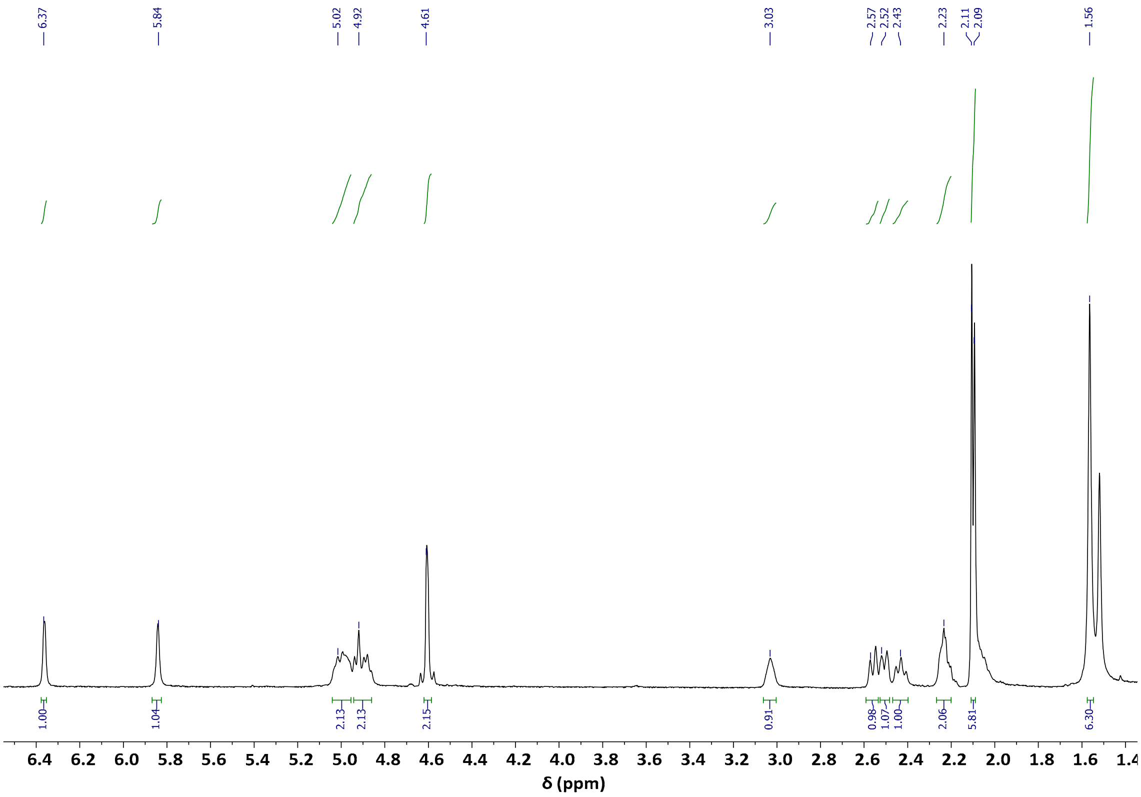

Figure S9. ^{1}H NMR spectrum of 8,15-*O,O′*-diacetylsalonitenolide (**5**) recorded in $CDCl_3$ at 400 MHz.

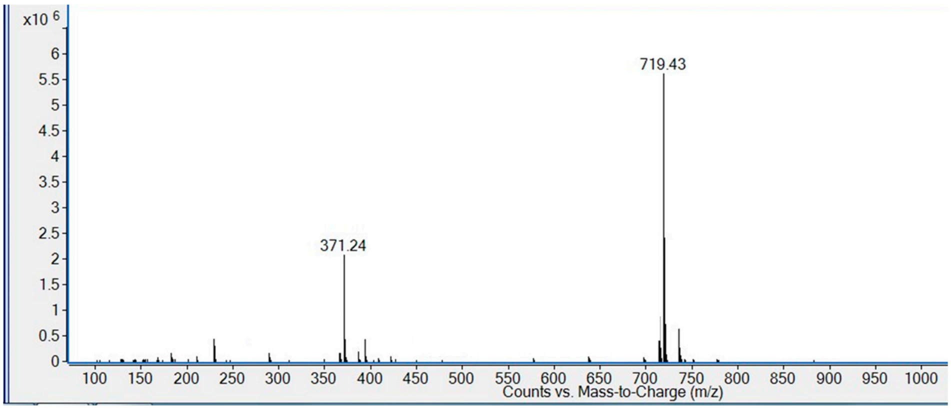

Figure S10. ESI MS spectrum of 8,15-*O,O′*-diacetylsalonitenolide (**5**) recorded in positive mode.
